# Developing combination strategies using PD-1 checkpoint inhibitors to treat cancer

**DOI:** 10.1007/s00281-018-0714-9

**Published:** 2018-10-29

**Authors:** Emmett V. Schmidt

**Affiliations:** Merck & Co., Inc., Kenilworth, NJ USA

**Keywords:** Immune checkpoint inhibitor, Pembrolizumab, PD-1, PD-L1, Independent action

## Abstract

More than 3000 clinical trials are evaluating the clinical activity of the PD-1 checkpoint inhibitors as monotherapies and in combinations with other cancer therapies [1]. The PD-1 checkpoint inhibitors are remarkable for their clinical activities in shrinking tumors across a wide range of tumor types, in causing durable responses, and in their tolerability. These attributes position them as favorable agents in clinical combinations. Historically, approaches to cancer therapy combinations focused on agents with orthogonal activities to avoid shared resistance mechanisms and shared toxicities. Although CTLA-4/PD-1 combinations have progressed based on possible immune interactions, additional approaches have used more orthogonal treatments such as standard of care chemotherapies and anti-angiogenesis inhibitors. Using the concept of independent activity pioneered by Bliss [2], examples of these approaches were compared. Both standard of care chemotherapy and anti-angiogenesis combinations show promising clinical activity above that predicted by the independent contributions of the agents tested on their own. In contrast, the combinations of CTLA4/PD-1 checkpoint inhibitors in renal cancer and melanoma show no more activity than that predicted by the independent contributions of the monotherapies. This update on approaches to the development of clinical combination therapies highlights the potential importance of combining PD-1 checkpoint inhibitors with a broad range of clinically active partners.

**Electronic supplementary material:**

The online version of this article (10.1007/s00281-018-0714-9) contains supplementary material, which is available to authorized users.

## Introduction

The PD-1 checkpoint inhibitors provide remarkable benefits for patients suffering from advanced cancers. As of August 2018, pembrolizumab has the broadest label among the PD-1 inhibitors and is indicated for the treatment of melanoma, non-small cell lung cancer (NSCLC), head and neck squamous cell cancer (HNSCC), classical Hodgkin Lymphoma (cHL), urothelial cancer, microsatellite instability-high cancers, gastric cancer, primary mediastinal B cell lymphoma, and cervical cancer.

Pembrolizumab and nivolumab were the fifth and sixth immunotherapeutics approved for the treatment of advanced melanoma (Table 1). The clinical activity of these drugs is remarkable in the context of cancer drug development. The overall response rate (ORR) in phase 1 for novel agents is generally predictive for subsequent regulatory approval in oncology (Table 1) [3, 4]. Thus, it is noteworthy that the PD-1 checkpoint inhibitors demonstrated a substantial increase in response rates compared with other immunotherapeutic agents [5, 6]. By this measure, these are the most active immunotherapeutic agents yet studied.

**Table 1:** Clinical activity as measured by overall response rates is associated with success in registration trials

A: Immunotherapeutic approvals in melanoma					
Product	Name	Approval	Indication	ORR	Reference
Proleukin	Interleukin 2	1992	Renal carcinoma	6%	[61]
Intron A	Interferon alfa-2b	2001	Adjuvant melanoma	8%	[62]
Sylatron	Peginterferon alfa-2b	2011	Adjuvant melanoma	6%	[63]
Yervoy	Ipilimumab	2011	Advanced melanoma	10.9%	[64]
Keytruda	Pembrolizumab	2014	Advanced melanoma	33%	[5]
Opdivo	Nivolumab	2014	Advanced melanoma	40%	[6]
Imlygic	T-Vec	2015	Advanced melanoma	16.3%	[65]
B: Association of ORR with drug approvals 1976–1993					
ORR (%)	Drugs 1976–1993	Trials that registered 1976–1993	Trials (%) 1976–19,932	Registration success (%) 1976–1993	Reference
0	59	10	33.9%	16.9%	[3]
0.1–5.0	64	14	36.8%	21.9%	
5.1–10	32	12	18.4%	37.5%	
>10	19	12	10.9%	63.2%	
C: Association of ORR with drug approvals 1985–1999					
Tumor type and response rate categories (%)	Total number of drugs	Number of drugs approved for any type of tumor (P)	Registration success (%) 1985–1999		Reference
0	8	1	12.5%		[4]
>0 and ≤ 10	20	0	0.0%		
>10 and ≤ 20	12	6	50.0%		
> 20	6	4	66.7%		

The promise of the new PD-1 checkpoint immunotherapies goes beyond their remarkable response rates. They offer a novel breadth of activity across indications, significant durability of response carrying over to survival benefit, and their manageable adverse event profiles facilitate combination therapy.

### Salient features of PD-1 checkpoint inhibition

#### The cellular dynamics of tumor shrinkage after release of checkpoint inhibition

PD-1 inhibitors release CD8 cells from immune checkpoint blockade, which then act as a remarkable cytotoxic machine to shrink tumors. Tumors are typically diagnosed when patients have a burden of cancer greater than 10^10^ cells [7]. Humans are thought to have 4 × 10^11^ circulating T cells [8] and the average clonotype targeting any specific antigen is thought to be in the range of 10 cells [9]. Since the release of CD8 cells from checkpoint inhibition by PD-1 therapies results in rapid tumor shrinkage [10], it seems reasonable to assume that anti-tumor T cells present at the initiation of PD-1 therapies are critical to the initial tumor response.

The very earliest reports of PD-1 efficacy in melanoma demonstrated remarkable outcomes for those patients who experienced complete responses [10, 11]. Spider plots in early papers showed that PD-1 treatment can induce a complete response in as few as 80 days, and a substantial fraction of the patients shown in the spider plots achieved partial responses in that time. Thus, while a tumor doubling time of 50 days, together with a starting cell mass containing 5 × 10^11^ cells implies that stable disease requires the killing of 1 × 10^10^ cells per day, a partial response will require the killing of 3 × 10^9^ more cells per day, and a complete response an additional 5 × 10^9^ cells per day. By any measure, the activation of CD8 tumor cell killing puts PD-1 inhibitors among the most cytotoxic of cancer therapies. Assuming a tumor doubling time of 50 days and the ability of a cytotoxic T cell to kill 2–16 cells per day [12], just to balance immune killing with tumor growth to achieve stable disease will require active killing by about 5 × 10^9^ CD8 effector cells, or about 1 in 100 of circulating T cells. This abundance is within range of detection by current sequencing technologies [13], which have been used to identify expansion of high frequency clones after initiation of PD-1 therapies in melanoma patients [14].

No other immunotherapy has been identified that is capable of this degree of tumor cell killing, highlighting this salient feature of the PD-1 inhibitors.

#### Breadth of activity

**Fig. 1:**
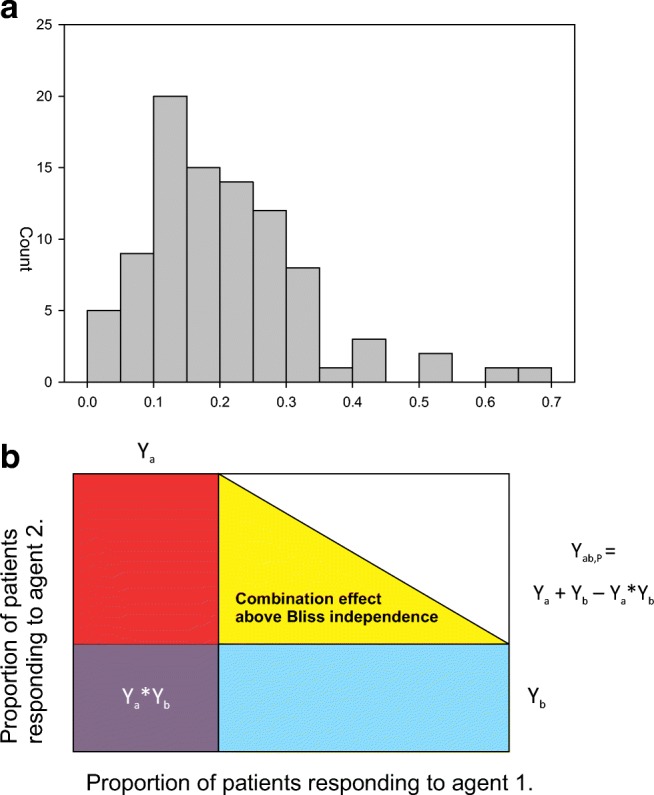
**a** Histogram showing the overall response rate for PD-1 checkpoint inhibitors used in monotherapy trials. Ninety trials with published ORRs for PD-1 checkpoint inhibitors across 31 indications were identified (references in supplementary materials). These studies also range across all lines of therapy and many were enriched by selection for PD-L1 tumor biomarker expression. These were plotted in a histogram with the x axis depicting the ORR for the trials and the y axis the numbers of trials demonstrating activity at the designated ORR. **b** The Bliss independent combination. The equation for Bliss independence is shown (Bliss, 1939). The square diagram shows conceptually that a drug with a Y_a_ response rate would combine with a second drug with a Y_b_ response rate, but the combined activity would not be expected to total just the two numbers. Rather their total must be corrected by their random interaction (Y_a_ × Y_b_), if their interaction is truly independent. x and y axes are a theoretic depiction of the range of possible response rates from 0 to 100% for each drug in a combination

Among the key initial studies of PD-1 and its ligand(s) PD-L1 (B7-H1) and PD-L2, the Chen laboratory demonstrated abundant expression of PD-L1 on a remarkably broad range of tumors [15]. As initial studies of PD-1 therapy in melanoma progressed, MSD Research Laboratories’ Keynote studies 012, 028, and 158 consequently sought to systematically explore the potential for pembrolizumab to work across a broad spectrum of tumors. In addition to studies of pembrolizumab, the activity of PD-1 and PD-L1 inhibitors has been reported for about 30 tumors types as of August 2018. The breadth of activity in multiple tumor types uncovered by these studies is among the many remarkable characteristics of these inhibitors (Fig. 1a). Figure 1a presents a histogram of ORRs published for 91 published monotherapy studies in 30 tumor types using the five approved PD-1 and PD-L1 inhibitors. The mean ORR for all of these studies is 20.7% (C.I. 1.3–25.8%) and the median is 18.7%. (This snapshot contains redundant data since the same agent has been tested in multiple lines of therapy, and different agents have been tested in the same indications.) In many cases, enrichment using PD-L1 biomarker strategies will have elevated the response rates, so this histogram is skewed toward higher response rates partly attributable to enrichment designs. Importantly, a trend toward greater efficacy in earlier lines of therapy can be found by comparing these data to the ORR for first line trials only. For the six tumor types where a PD-1 or PD-L1 has been studied as a first line therapy in an all comer’s population, the mean ORR improves to 31.3% (C.I. 27.9–59.1%) and the median ORR is 26%. Nevertheless, this breadth of activity has not been seen for any other immunotherapeutic, and the predictive value of these ORRs for eventual success in regulatory approvals [3, 4] suggests that a PD-1 or PD-L1 inhibitor will likely become part of the treatment paradigm for nearly all cancer types in the future.

#### Duration of PD-1 treatment effect

In contrast to chemotherapies or targeted therapies, immunotherapies offer a unique promise for longer term efficacy. By re-activating the CD8 cell response that is presumed to have become “exhausted” before diagnosis, the PD-1 therapies induce a durable immune clearance of tumors. Overall survival data support that view. The 5-year overall survival (OS) rate in Keynote 001 with pembrolizumab treatment for treatment-naive melanoma patients is now 41% [16]. The 4-year OS for NSCLC patients initially treated with pembrolizumab is 27%. These numbers appear to be plateauing after 42 months. More remarkable, these effects may not require lifelong treatment with PD-1 inhibitors. The overall survival rate for 67 melanoma patients who had complete responses to pembrolizumab, discontinued PD-1 therapy, and received no further therapy was 89.9% [17]. To further assess the need for long-term treatment, in Keynote 006 pembrolizumab was prospectively discontinued after completion of 2 years of therapy [18]. With 46 months of follow up, the 4-year OS for these patients is 42%, similar to the results in Keynote 001 where patients were treated to progression. After planned discontinuation of the checkpoint inhibitor, with a median follow-up of 21 months, 86% of patients remain without disease progression off therapy. These statistics highlight the potential for checkpoint inhibitors to have re-programmed immune responses to tumors in the direction of long-term benefit well after completion of treatment courses.

#### Manageable adverse event profile

The classic drug development approach of combining therapies that function through orthogonal mechanisms works not only to prevent development of single overlapping mechanism resistance, but also avoids exacerbating overlapping toxicities [19]. The toxicities of immune checkpoint inhibitors are inherent in their mechanism, which was immediately obvious when the PD-1 and PD-L1 gene targets were identified [20]. Disruption of the PD-1 gene causes a lupus like autoimmune disease in mice. While this association was readily apparent in animal models, genetic associations between PD-1 gene polymorphisms and autoimmunity remain somewhat elusive in humans [21, 22]. On the other hand, side effects of checkpoint inhibitors fit the obvious expected autoimmune patterns predicted by the genetic models [23]. PD-1 checkpoint inhibitor side effects most frequently involve the endocrine glands, skin, gastrointestinal tract, liver, and lungs.

Meta-analyses have shown that checkpoint inhibitor-related risk for hypothyroidism, pneumonitis, colitis, and hypophysitis is increased compared with control treatments [24]. These meta-analyses also demonstrate non-overlapping toxicities of checkpoint inhibitors when compared with the side effects of chemotherapies and targeted therapies. In contrast, combinations of PD-1 and CTLA 4 inhibitors result in higher rates of specific shared adverse events consistent with synergistic toxicity [25]. Rash, pruritus, and diarrhea all increase in frequency in CTLA4/PD-1 combined therapy. Increased ALTs, colitis, hypophysitis, hypothyroidism, and pneumonitis occur in these combinations, and can be dose-limiting and life threatening. A healthy debate continues regarding the benefit/risk ratio of combining checkpoint inhibitors. However, the historic approach to cancer combinations using independently acting compounds would argue for combination therapies that do not include overlapping immune toxicities.

### What is to be done?

Despite high response rates to PD-1/L1 monotherapy, Fig. 1 underscores the unmet medical need of patients not responding to checkpoint inhibition. At this time, less than half of patients will respond to monotherapy treatment with checkpoint inhibitors. This unmet need could be approached either by using biomarkers for personalized treatments, or by identifying effective combination therapies.

### Biomarkers

This review will focus on combination strategies for immune checkpoint inhibitors. However, the ligand receptor pairing of PD-1 provides a strong rationale for PD-L1 as a predictive biomarker, which has been a highly successful approach to bringing PD-1 checkpoint inhibitors to patients most likely to benefit from their use. Exploiting its expression on tumor cells, a companion diagnostic has been approved by the FDA to identify patients with NSCLC, bladder, gastric, head, and neck squamous cell and cervical cancers likely to benefit from treatment with pembrolizumab [26]. Several other PD-L1 assays are available for use with other PD-1 inhibitors [27]. Unlike many gene tests, PD-L1 expression varies across a gradient in immunohistochemistry assays, requiring the development of empiric cut points derived from receiver operator curves to optimize assay use. A variety of exploratory assays in development evaluate T cell receptor clonality, tumor infiltrating lymphocytes, mutational burden, immune gene signatures, and multiplex immunohistochemistry [28]. These assays will similarly require clinical correlation to identify cut points since they also measure continuous variables. Complexities of the assay platforms used for these additional assays seem likely to slow or inhibit their use at this time compared with the success of PD-L1 as a companion diagnostic.

### Tumor size, resistance to therapy, and rationale for combining agents

We have entered a new era in the development of the checkpoint inhibitors where development of more effective combination regimens will be critical to further progress.

Basic principles of cancer combination therapy were developed in the first decade of use of chemotherapeutic agents [29]. In a seminal analysis, Law articulated fundamental principles [30]. First, therapeutic selectivity for cancer cells versus normal cells is fundamental to the agents used and to their use in combination. Second, evolution of resistance has always been proposed as the major contributor to eventual treatment failure. The development of immunotherapeutic combinations may now be coming to better understand these basic principles [31]. The manageable side effects of PD-1 checkpoint inhibitors readily address the first principle [32].

The nature of resistance to PD-1 checkpoint inhibitors is only now progressing as a topic for investigation [33]. However, in contrast to the sophisticated molecular tools being used to identify resistance mechanisms, a consideration of simpler approaches to understanding resistance may be helpful as well. Law’s initial work observed that mutation rates are fundamental to the development of resistance. It is simple to note that the probability of resistance emerging from combinations of agents with different and independent mechanisms is the product of the frequency of resistance mutations for each of the agents. This is one powerful argument in favor of combining agents with orthogonal mechanisms of action.

DeVita and colleagues further linked resistance to cancer therapy to tumor mass following simple logic [7]. Resistance will be tumor mass related simply because the likelihood of a resistant clone developing is related to the cell numbers in a tumor. This logic was further linked to Law’s principles of combination therapy to infer that multiple agents shrinking tumors by any mechanisms could combine successfully. These simple principles should be no less true for immunotherapy. If preexisting anti-tumor T cell repertoires are fundamental to the success of PD-1 therapies, and if those repertoires are limited, then the ratio of tumor targeted T cells to the net number of tumor cells will be critical to the success of PD-1 treatments. Accordingly, the role of tumor burden in immunotherapeutic resistance was recently shown by the Wherry lab [34]. So as anticipated by Devita’s classic arguments, any agent that shrinks tumors will bring the net number of tumor cells within range of the effects of PD-1 activation of the CD8 tumor killing response.

## Drivers in the clinical development of cancer combination therapy

### The nature of combination effect

In their prospective thinking scientists often seem susceptible to “latency bias,” despite the rigor of the scientific method. Just as physicians can be trapped into approaching their next case with an eye to their immediately preceding one, the scientific community can find itself looking at new paradigms using thinking guided by any immediately preceding paradigm shift. The clinical cancer community finds itself in a transition from the era of novel targeted therapies to the era of immune oncologic agents. Given the success in developing BRAF/MEK combination therapies based on the identification of molecular resistance mechanisms, it is not surprising to see an immediate focus on the identification of resistance mechanisms to the PD-1 checkpoint inhibitors as a paradigm to develop rational combinations [33]. With that approach however, “synergy” may be in danger of becoming more of a biological model than a drug combination concept.

It is worth noting that considerations of the combined action of therapeutic agents have been with us for nearly 100 years [35]. By simple logic, the mathematic interaction between combination agents should start with the determination of whether both agents are individually effective, only one of them is effective, or neither is effective when combined. A rigorous mathematic definition should then more carefully attribute a combination to “synergy” if the combination effect is greater than the mathematic probability of the two agents contributing independently (synergy), equal to the probability of their independent activities (additivity) or less than predicted (antagonism). Additivity when only one agent is active has also been described as inertism.

#### Independent contributions to combined action

The algebra of independent contribution is attractively simple [2]. Simply by probability, the combined effect of independently active agents should be:

$$ {\mathrm{Y}}_{\mathrm{a}\mathrm{b},\mathrm{P}}={\mathrm{Y}}_{\mathrm{a}}+{\mathrm{Y}}_{\mathrm{b}}\hbox{--} {\mathrm{Y}}_{\mathrm{a}}\times {\mathrm{Y}}_{\mathrm{b}} $$

This is often envisioned by a simple box diagram (Fig. 1b), where the box encompasses the responses of a group of patients, a horizontal rectangle identifies the patients responding to one agent, and a vertical rectangle identifies those responding to the other. The equation is a test of independence since the combined response rate should be less than the simple sum if the drugs have no interaction.

#### Achieving Lowe additivity

“Synergy” as a goal becomes more complex if the goal is to achieve more from a combination than either agent can contribute on their own. The BRAF-MEK model provides a view of the potential success of that goal in a biological sense. However, clinical synergy is often confused by the aspirations of in vitro studies, with hopes of predicting synergy in the clinic. The in vitro field is substantially more complex than clinical development since it is possible to identify true synergy through the use of response surfaces like those of Chou and Talaly [36]. Since it is rare in clinical practice, and especially in clinical oncology, to be willing to sacrifice the full effect of either of two agents if used at sub therapeutic doses, the evaluation of surface response interactions is rare to nonexistent in clinical practice. In contrast, Palmer and Sorger recently applied the principles of Bliss independence to analyze the contributions of ipilimumab and nivolumab to their combination efficacy in treating advanced melanoma [31]. They applied Bliss independence predictions to measures of overall tumor shrinkage (Waterfall plots) and progression-free survival (PFS) to argue for the absence of rigorous mathematical synergy in that combination despite broad claims for biological synergy. PFS has proven to be a less than ideal endpoint to measure the clinical effect of immune oncology agents, often failing to show an effect where the ORR coupled with durability of response, and overall survival more clearly measure the benefit of immunotherapeutics to patients. Using several case studies, this review will assess contributions of combinations of PD-1 therapies for a subset of the most promising combinations to better understand the potential to obtain greater benefit than that achieved independently by either agent or regimen alone.

## Case studies in PD-1 clinical combination development

Remarkable advances have been seen in the last 2 years combining PD-1 checkpoint inhibitors with standard of care agents, angiogenesis inhibitors, and by combining more than one checkpoint inhibitor (Table 2).

**Table 2:** Clinical combinations including PD-1 checkpoint inhibitors

PD-1	Combination agent	Indication	Combination ORR	ORR combination agent	PD-1 monotherapy ORR^1^	Bliss independence prediction	Additional contribution of combination (Z^2^)
Pembrolizumab	Pemetrexed-carboplatin	NSCLC	71% [39]	31% [66]	23% [67]	54%	0.17
Pembrolizumab	Paclitaxel-carboplatin	NSCLC	52% [39]	15% [68]	23% [67]	38%	0.14
Pembrolizumab	Paclitaxel-carboplatin-bevacizumab	NSCLC	48% [39]	35% [68]	23% [67]	58%	− 0.10
Nivolumab	Paclitaxel-carboplatin	NSCLC	47% [41]	15% [68]	23% [67]	38%	0.09
Nivolumab	Pemetrexed-cisplatin	NSCLC	47% [41]	32% [69]	23% [67]	55%	− 0.08
Nivolumab	Gemcitabine-cisplatin	NSCLC	33% [41]	30% [69]	23% [67]	53%	− 0.20
Pembrolizumab	Axitinib	RCC	73% [52]	19% [50]	25% [48]	44%	0.29
Pembrolizumab	Lenvatinib	RCC	67% [53]	19% [51]	25% [48]	44%	0.23
Nivolumab	Ipilimumab	RCC	42% [70]	13% [47]	38% [49]	38%	0.04
Nivolumab	Ipilimumab	Melanoma	58% [58]	11% [64]	40% [6]	51%	0.07

^1^The PD-1 monotherapy ORR used in the Bliss calculation corresponds to the available ORR for any of the PD-1s in the corresponding line of therapy, given the increase in ORR seen in earlier lines of therapy. If the ORR is known in the same line of therapy for more than one PD-1, the monotherapy ORR used corresponds to the PD-1 in the combination where possible

^2^The derived equation for any additional combination contribution is Z = ORR − (Y_a_ + Y_b_ – Y_a_ × Y_b_). Z can be either positive or negative, depending on whether the Bliss independent prediction is less than or greater than the measured ORR for a combination

### Standard of care chemotherapeutics

The empiric combination of newly emerging agents with standard of care therapies has moved oncology forward since the chemotherapy era began [37]. Platinum-based chemotherapy doublets were established as a standard of care for the first line treatment of NSCLC prior to the emergence of PD-1 therapies [38]. Since carboplatin + paclitaxel, carboplatin + paclitaxel + bevacizumab, and carboplatin + pemetrexed were established standards, pembrolizumab was compared as a combination agent for each of those three regimens [39]. A complex but steady line of experimentation has also proposed immunologic contributions of standard chemotherapy to their clinical activity [40], and these experiments provide evidence that some chemotherapy is less effective in immune deficient animal models. Thus, these combinations had the potential to benefit both from independent activity as well as beneficial immune interactions. A similar trial combined nivolumab with gemcitabine + cisplatin, pemetrexed + cisplatin, paclitaxel + carboplatin, and paclitaxel + carboplatin [41]. In Table 2, the components of the Bliss independent contributions to the total combination ORR were identified and calculated for these six combination studies in initial phase 1 trials. From this simple calculation, it is evident that the independent contributions of the agents might predict a large portion of the combination ORR, and that an additional contribution can be seen as well. The ORR of these agents in combination exceeded the ORR predicted by the Bliss equation [2]. These calculations should be considered measures of central tendency given the limitations of phase 1–2 data. With the small numbers of subjects involved, which preclude controlling for variability, these conclusions should be considered directional. Nevertheless, this framework to evaluating clinical data offers an objective approach to the assessment of combination effects.

By subtracting the Bliss prediction from the actual combination ORR, an additional combination specific contribution (Z) can be assessed. This calculation was not anticipated in the original Bliss publication, but is offered here as a further objective measure of what might be a “synergistic” effect that can be calculated objectively in a phase 1 proof of concept clinical trial. Since this value is most positive for the pemetrexed-platinum combinations, the clinical trial evidence here supports a long line of investigations evaluating the immunological effects of platinum therapies [42].

Limits to the interpretation of phase 1 trials are well known. Their small numbers limit statistical rigor, and the typical use of single arm structures precludes rigorous comparisons. Importantly, however, the overall clinical benefit of this combination has been fully supported by the randomized trials that followed. One hundred twenty-three patients were enrolled in Keynote 21G, a randomized comparison between the standard of care pemetrexed-platinum doublet and the doublet with pembrolizumab added, and the trial’s primary endpoint of an increase in the ORR from the standard of care to the triplet combination was achieved showing an increase from 29% (C.I. 18–41) to 55% (C.I. 42–68) [43]. These responses were quite durable, since the median duration of response was not reached in the initial publication. These phases 1 and 2 proof of concept studies were followed by a large randomized phase 3 trial, Keynote 189 [44]. Six hundred sixteen patients were randomized 2:1 to receive the pembrolizumab containing triplet combination versus the platinum-pemetrexed doublet. Primary endpoints of overall survival and progression-free survival were both met with hazard ratios of 0.49 (C.I. 0.38–0.64) and 0.52 (C.I. 0.43–0.62). While starting from the simple principle of adding a new agent to the current standard of care, supported by rigorous early signal finding studies, this randomized phase 3 trial established a new standard of care for first line lung cancer [45], and it now offers a hypothesis generating clinical data set to explore the postulated immune oncology contributions of platinum-based chemotherapy.

### Angiogenesis inhibition

Vascular endothelial growth factor (VEGF) and vascular endothelial growth factor receptor (VEGFR) inhibitors are effective standard of care agents used in the treatment of renal cancer [46]. The logic to combine with PD-1 checkpoint inhibitors readily follows the earlier “add-on” logic in lung cancer. These combinations are moving rapidly through phases 1 and 2 programs, and the results of randomized phase 3 trials may occur by the time of publication of this review. For comparison, early in the development of CTLA4 inhibitors, Rosenberg and colleagues evaluated ipilimumab monotherapy in the treatment of renal cancer [47]. They demonstrated a response rate of 13% to ipilimumab in second line treatment. PD-1 therapies have now been tested as monotherapies as both second and first line treatments for renal cell carcinoma with nivolumab showing a 25% response rate in the second line [48] and pembrolizumab demonstrating a 38% (C.I. 29–48) response rate in first line RCC [49]. Axitinib [50] and lenvatinib [51] are advanced VEGFR TKIs with favorable activity in renal cell carcinoma. Combinations of pembrolizumab with axitinib [52] and pembrolizumab with lenvatinib [53] show promising phase 1–2 combination activity. The ORRs for these combinations are shown in Table 2, along with the ORR for the combination of nivolumab and ipilimumab [54]. Like the platinum and PD-1 combination in lung cancer, the combination of a PD-1 inhibitor to standard of care angiogenesis inhibitors shows a combination ORR that apparently benefits not only from a Bliss independent contribution of both components, but also shows an additional contribution related to the combination itself (Z). A Bliss independent model calculation for the combination of ipilimumab and nivolumab shows that ipilimumab as the combining agent achieves no more combination activity than predicted by the Bliss model. It is additionally interesting to see that any combination specific contribution (z) for ipilimumab and nivolumab falls below the calculations for the two VEGFR inhibitors. As for the platinum therapies, a long history of studies of VEGFR inhibitors has demonstrated their ability to interact with the immune system [55]. Here again, starting with more orthogonal combination partners, the combination ORRs show more than a Bliss additive effect, lending objective clinical evidence to support previous studies of immune effects of the VEGFR inhibitors.

### Dual checkpoint inhibition in melanoma

The concept to combine two checkpoint inhibitors was initially demonstrated using murine model systems [56]. The development of the ipilimumab and nivolumab combination in melanoma from early phase studies [57] to phase 3 registration trials [58] was based on this immunologic reasoning. As described above, Palmer and Sorger failed to find a contribution of the combination for progression-free survival beyond that predicted by the independent contributions of the two agents taken on their own. To further assess the role of independent action, Table 2 further shows an analysis of the ORRs for the ipilimumab and nivolumab combination in melanoma, which again shows that much of its combination ORR can be attributed to independent action. Notably, a trial sequencing these agents by immunologic reasoning [59] failed to match immunologic predictions for the functions of these two agents [60] so even that reasoning may require continued evaluation.

## Conclusion

A recent compilation of the landscape of clinical trials testing novel immunotherapies identified 3042 active registered clinical trials at the end of 2017 [1]. A large proportion of those studies involve combinations of PD-1 checkpoint inhibitors with a broad range of additional agents. The scale of these studies makes it hard to summarize their intent and results easily. Nevertheless, this brief review sought to highlight the logic behind three combination paradigms, and their emerging results. Indeed, the Tang review showed that CTLA4-PD-1, angiogenesis inhibitor-PD-1, and chemotherapy-PD-1 combinations are the leading combination hypotheses in trials at this time. Using the Bliss model for independent activity of two agents or regimens in combination, combinations involving chemotherapy and angiogenesis inhibitor both show more than independent combination activity. In contrast, the combination of two checkpoint inhibitors in renal cancer and melanoma shows no more activity than expected for the independent contribution of the two agents. This review, and these principles, suggests that there is a promising future for a broad range of possible combinations of PD-1 checkpoint inhibitors with other cancer therapies.

## Electronic supplementary material

ESM 1 (DOCX 140 kb)
