# Supplementary material for: Developing combination strategies using PD-1 checkpoint inhibitors to treat cancer
Source: Semin Immunopathol. 2018 Oct 29;41(1):21–30. doi: 10.1007/s00281-018-0714-9 (PMC6323091; doi:10.1007/s00281-018-0714-9)
Supplement: Supplementary file 1 — (DOCX 140 kb) [file 281_2018_714_MOESM1_ESM.docx]

## Supplementary references to Figure 1A.

[[1](#_ENREF_1)], [[2](#_ENREF_2)], [[3](#_ENREF_3)], [[4](#_ENREF_4)], [[5](#_ENREF_5)], [[6](#_ENREF_6)], [[7](#_ENREF_7)], [[8](#_ENREF_8)], [[9](#_ENREF_9)], [[10](#_ENREF_10)], [[11](#_ENREF_11)], [[12](#_ENREF_12)], [[13](#_ENREF_13)], [[14](#_ENREF_14)], [[15](#_ENREF_15)], [[16](#_ENREF_16)], [[17](#_ENREF_17)], [[18](#_ENREF_18)], [[19](#_ENREF_19)], [[20](#_ENREF_20)], [[21](#_ENREF_21)], [[22](#_ENREF_22)], , [[23](#_ENREF_23)], [[24](#_ENREF_24)], [[25](#_ENREF_25)], [[26](#_ENREF_26)], [[27](#_ENREF_27)], [[28](#_ENREF_28)], [[29](#_ENREF_29)], [[30](#_ENREF_30)], [[31](#_ENREF_31)], [[32](#_ENREF_32)], [[33](#_ENREF_33)], [[34](#_ENREF_34)], [[35](#_ENREF_35)], [[36](#_ENREF_36)], [[37](#_ENREF_37)], [[38](#_ENREF_38)], [[39](#_ENREF_39)], [[40](#_ENREF_40)], [[41](#_ENREF_41)], [[37](#_ENREF_37)], [[42](#_ENREF_42)], [[43](#_ENREF_43)], [[44](#_ENREF_44)], [[45](#_ENREF_45)], [[46](#_ENREF_46)], [[47](#_ENREF_47)], [[37](#_ENREF_37)], [[48](#_ENREF_48)], [[49](#_ENREF_49)], [[50](#_ENREF_50)], [[51](#_ENREF_51)], [[52](#_ENREF_52)], [[53](#_ENREF_53)], [[54](#_ENREF_54)], [[55](#_ENREF_55)], [[56](#_ENREF_56)], [[57](#_ENREF_57)], [[58](#_ENREF_58)], [[59](#_ENREF_59)], [[60](#_ENREF_60)], [[61](#_ENREF_61)], [[62](#_ENREF_62)], [[63](#_ENREF_63)], [[64](#_ENREF_64)], [[65](#_ENREF_65)], [[64](#_ENREF_64)], [[66](#_ENREF_66)], [[67](#_ENREF_67)], [[68](#_ENREF_68)], [[69](#_ENREF_69)], [[70](#_ENREF_70)], [[70](#_ENREF_70)], [[71](#_ENREF_71)], [[72](#_ENREF_72)], [[73](#_ENREF_73)], [[74](#_ENREF_74)], [[75](#_ENREF_75)], [[75](#_ENREF_75)], [[76](#_ENREF_76)], [[65](#_ENREF_65)], [[77](#_ENREF_77)], [[32](#_ENREF_32)], [[32](#_ENREF_32)], , [[77](#_ENREF_77)], [[78](#_ENREF_78)], [[79](#_ENREF_79)],

[1] R. Chen, P.L. Zinzani, M.A. Fanale, P. Armand, N.A. Johnson, P. Brice, J. Radford, V. Ribrag, D. Molin, T.P. Vassilakopoulos, A. Tomita, B. von Tresckow, M.A. Shipp, Y. Zhang, A.D. Ricart, A. Balakumaran, C.H. Moskowitz, Keynote, Phase II Study of the Efficacy and Safety of Pembrolizumab for Relapsed/Refractory Classic Hodgkin Lymphoma, J Clin Oncol 35(19) (2017) 2125-2132.

[2] S.P. D'Angelo, J. Russell, C. Lebbe, B. Chmielowski, T. Gambichler, J.J. Grob, F. Kiecker, G. Rabinowits, P. Terheyden, I. Zwiener, M. Bajars, M. Hennessy, H.L. Kaufman, Efficacy and Safety of First-line Avelumab Treatment in Patients With Stage IV Metastatic Merkel Cell Carcinoma: A Preplanned Interim Analysis of a Clinical Trial, JAMA oncology (2018).

[3] R. Chen, A.L. Gibb, G.P. Collins, R. Popat, D. El-Sharkawi, C. Burton, D. Lewis, F.M. Miall, A. Forgie, A. Compagnoni, G. Andreola, S. Brar, A. Thall, A. Woolfson, J. Radford, BLOCKADE OF THE PD-1 CHECKPOINT WITH ANTI–PD-L1 ANTIBODY AVELUMAB IS SUFFICIENT FOR CLINICAL ACTIVITY IN RELAPSED/REFRACTORY CLASSICAL HODGKIN LYMPHOMA (CHL), Hematological oncology 35(S2) (2017) 67-67.

[4] P. Nghiem, S. Bhatia, E.J. Lipson, W.H. Sharfman, R.R. Kudchadkar, P.A. Friedlander, A.S. Brohl, A. Daud, H.M. Kluger, S.A. Reddy, M.A. Burgess, B.A. Hanks, T. Olencki, B.C. Boulmay, L.M. Lundgren, N. Ramchurren, B.H. Moreno, E. Sharon, M.A. Cheever, S.L. Topalian, C.-t. group, Durable tumor regression and overall survival (OS) in patients with advanced Merkel cell carcinoma (aMCC) receiving pembrolizumab as first-line therapy, Journal of Clinical Oncology 36(15_suppl) (2018) 9506-9506.

[5] P.L. Zinzani, V. Ribrag, C.H. Moskowitz, J.-M. Michot, J. Kuruvilla, A. Balakumaran, E. Snyder, P. Marinello, M.A. Shipp, P. Armand, Phase 1b Study of PD-1 Blockade with Pembrolizumab in Patients with Relapsed/Refractory Primary Mediastinal Large B-Cell Lymphoma (PMBCL), Blood 126(23) (2015) 3986-3986.

[6] C. Robert, G.V. Long, B. Brady, C. Dutriaux, M. Maio, L. Mortier, J.C. Hassel, P. Rutkowski, C. McNeil, E. Kalinka-Warzocha, K.J. Savage, M.M. Hernberg, C. Lebbe, J. Charles, C. Mihalcioiu, V. Chiarion-Sileni, C. Mauch, F. Cognetti, A. Arance, H. Schmidt, D. Schadendorf, H. Gogas, L. Lundgren-Eriksson, C. Horak, B. Sharkey, I.M. Waxman, V. Atkinson, P.A. Ascierto, Nivolumab in previously untreated melanoma without BRAF mutation, N Engl J Med 372(4) (2015) 320-30.

[7] C. Robert, A. Ribas, O. Hamid, A. Daud, J.D. Wolchok, A.M. Joshua, W.-J. Hwu, J.S. Weber, T.C. Gangadhar, R.W. Joseph, R.S. Dronca, A. Patnaik, H.M. Zarour, R. Kefford, P. Hersey, X. Li, S.J. Diede, S. Ebbinghaus, F.S. Hodi, Three-year overall survival for patients with advanced melanoma treated with pembrolizumab in KEYNOTE-001, Journal of Clinical Oncology 34(15_suppl) (2016) 9503-9503.

[8] D.F. McDermott, J.-L. Lee, C. Szczylik, F. Donskov, J. Malik, B.Y. Alekseev, J.M.G. Larkin, V.B. Matveev, R.A. Gafanov, P. Tomczak, S.S. Tykodi, P.F. Geertsen, P.J. Wiechno, S.J. Shin, F. Pouliot, T.A. Gordoa, W. Li, R.F. Perini, C. Schloss, M.B. Atkins, Pembrolizumab monotherapy as first-line therapy in advanced clear cell renal cell carcinoma (accRCC): Results from cohort A of KEYNOTE-427, Journal of Clinical Oncology 36(15_suppl) (2018) 4500-4500.

[9] L.A. Diaz, A. Marabelle, J.-P. Delord, R. Shapira-Frommer, R. Geva, N. Peled, T.W. Kim, T. Andre, E.V. Cutsem, R. Guimbaud, D. Jaeger, E. Elez, T. Yoshino, A.K. Joe, B. Lam, C.K. Gause, S.K. Pruitt, S.P. Kang, D.T. Le, Pembrolizumab therapy for microsatellite instability high (MSI-H) colorectal cancer (CRC) and non-CRC, Journal of Clinical Oncology 35(15_suppl) (2017) 3071-3071.

[10] Y.-J. Bang, H.-C. Chung, V. Shankaran, R. Geva, D.V.T. Catenacci, S. Gupta, J.P. Eder, R. Berger, E.J. Gonzalez, A. Ray, M. Dolled-Filhart, K. Emancipator, K. Pathiraja, J.K. Lunceford, J.D. Cheng, M. Koshiji, K. Muro, Relationship between PD-L1 expression and clinical outcomes in patients with advanced gastric cancer treated with the anti-PD-1 monoclonal antibody pembrolizumab (MK-3475) in KEYNOTE-012, Journal of Clinical Oncology 33(15_suppl) (2015) 4001-4001.

[11] H.L. Kaufman, J.S. Russell, O. Hamid, S. Bhatia, P. Terheyden, S.P. D'Angelo, K.C. Shih, C. Lebbe, M. Milella, I. Brownell, K.D. Lewis, J.H. Lorch, A. von Heydebreck, M. Hennessy, P. Nghiem, Updated efficacy of avelumab in patients with previously treated metastatic Merkel cell carcinoma after >/=1 year of follow-up: JAVELIN Merkel 200, a phase 2 clinical trial, Journal for immunotherapy of cancer 6(1) (2018) 7.

[12] P.A. Ott, E. Elez, S. Hiret, D.-W. Kim, A. Morosky, S. Saraf, B. Piperdi, J.M. Mehnert, Pembrolizumab in Patients With Extensive-Stage Small-Cell Lung Cancer: Results From the Phase Ib KEYNOTE-028 Study, Journal of Clinical Oncology 35(34) (2017) 3823-3829.

[13] C. Robert, J. Schachter, G.V. Long, A. Arance, J.J. Grob, L. Mortier, A. Daud, M.S. Carlino, C. McNeil, M. Lotem, J. Larkin, P. Lorigan, B. Neyns, C.U. Blank, O. Hamid, C. Mateus, R. Shapira-Frommer, M. Kosh, H. Zhou, N. Ibrahim, S. Ebbinghaus, A. Ribas, K.-. investigators, Pembrolizumab versus Ipilimumab in Advanced Melanoma, N Engl J Med (2015).

[14] J.S. Weber, S.P. D'Angelo, D. Minor, F.S. Hodi, R. Gutzmer, B. Neyns, C. Hoeller, N.I. Khushalani, W.H. Miller, Jr., C.D. Lao, G.P. Linette, L. Thomas, P. Lorigan, K.F. Grossmann, J.C. Hassel, M. Maio, M. Sznol, P.A. Ascierto, P. Mohr, B. Chmielowski, A. Bryce, I.M. Svane, J.J. Grob, A.M. Krackhardt, C. Horak, A. Lambert, A.S. Yang, J. Larkin, Nivolumab versus chemotherapy in patients with advanced melanoma who progressed after anti-CTLA-4 treatment (CheckMate 037): a randomised, controlled, open-label, phase 3 trial, Lancet Oncol (2015).

[15] G.V. Long, R. Dummer, O. Hamid, T. Gajewski, C. Caglevic, S. Dalle, A. Arance, M.S. Carlino, J.-J. Grob, T.M. Kim, L.V. Demidov, C. Robert, J.M.G. Larkin, J. Anderson, J.E. Maleski, M.M. Jones, S.J. Diede, T.C. Mitchell, Epacadostat (E) plus pembrolizumab (P) versus pembrolizumab alone in patients (pts) with unresectable or metastatic melanoma: Results of the phase 3 ECHO-301/KEYNOTE-252 study, Journal of Clinical Oncology 36(15_suppl) (2018) 108-108.

[16] T. Doi, S.A. Piha-Paul, S.I. Jalal, S. Saraf, J. Lunceford, M. Koshiji, J. Bennouna, Safety and Antitumor Activity of the Anti–Programmed Death-1 Antibody Pembrolizumab in Patients With Advanced Esophageal Carcinoma, Journal of Clinical Oncology 36(1) (2018) 61-67.

[17] R.S. Herbst, P. Baas, D.W. Kim, E. Felip, J.L. Perez-Gracia, J.Y. Han, J. Molina, J.H. Kim, C.D. Arvis, M.J. Ahn, M. Majem, M.J. Fidler, G. de Castro, Jr., M. Garrido, G.M. Lubiniecki, Y. Shentu, E. Im, M. Dolled-Filhart, E.B. Garon, Pembrolizumab versus docetaxel for previously treated, PD-L1-positive, advanced non-small-cell lung cancer (KEYNOTE-010): a randomised controlled trial, Lancet 387(10027) (2016) 1540-50.

[18] P.H. O'Donnell, E.R. Plimack, J. Bellmunt, R. Berger, R.B. Montgomery, K. Heath, M. Dolled-Filhart, K. Pathiraja, C.K. Gause, J.D. Cheng, R.F. Perini, S. Gupta, Pembrolizumab (Pembro; MK-3475) for advanced urothelial cancer: Results of a phase IB study, Journal of Clinical Oncology 33(7_suppl) (2015) 296-296.

[19] T. Powles, J.P. Eder, G.D. Fine, F.S. Braiteh, Y. Loriot, C. Cruz, J. Bellmunt, H.A. Burris, D.P. Petrylak, S.L. Teng, X. Shen, Z. Boyd, P.S. Hegde, D.S. Chen, N.J. Vogelzang, MPDL3280A (anti-PD-L1) treatment leads to clinical activity in metastatic bladder cancer, Nature 515(7528) (2014) 558-62.

[20] E.R. Plimack, J. Bellmunt, S. Gupta, R. Berger, L.Q. Chow, J. Juco, J. Lunceford, S. Saraf, R.F. Perini, P.H. O'Donnell, Safety and activity of pembrolizumab in patients with locally advanced or metastatic urothelial cancer (KEYNOTE-012): a non-randomised, open-label, phase 1b study, Lancet Oncol 18(2) (2017) 212-220.

[21] J.E. Rosenberg, J. Hoffman-Censits, T. Powles, M.S. van der Heijden, A.V. Balar, A. Necchi, N. Dawson, P.H. O'Donnell, A. Balmanoukian, Y. Loriot, S. Srinivas, M.M. Retz, P. Grivas, R.W. Joseph, M.D. Galsky, M.T. Fleming, D.P. Petrylak, J.L. Perez-Gracia, H.A. Burris, D. Castellano, C. Canil, J. Bellmunt, D. Bajorin, D. Nickles, R. Bourgon, G.M. Frampton, N. Cui, S. Mariathasan, O. Abidoye, G.D. Fine, R. Dreicer, Atezolizumab in patients with locally advanced and metastatic urothelial carcinoma who have progressed following treatment with platinum-based chemotherapy: a single-arm, multicentre, phase 2 trial, Lancet 387(10031) (2016) 1909-20.

[22] D.P. Petrylak, T. Powles, J. Bellmunt, F. Braiteh, Y. Loriot, R. Morales-Barrera, H.A. Burris, J.W. Kim, B. Ding, C. Kaiser, M. Fasso, C. O'Hear, N.J. Vogelzang, Atezolizumab (MPDL3280A) Monotherapy for Patients With Metastatic Urothelial Cancer: Long-term Outcomes From a Phase 1 Study, JAMA oncology 4(4) (2018) 537-544.

[23] D.P. Carbone, M. Reck, L. Paz-Ares, B. Creelan, L. Horn, M. Steins, E. Felip, M.M. van den Heuvel, T.E. Ciuleanu, F. Badin, N. Ready, T.J.N. Hiltermann, S. Nair, R. Juergens, S. Peters, E. Minenza, J.M. Wrangle, D. Rodriguez-Abreu, H. Borghaei, G.R. Blumenschein, Jr., L.C. Villaruz, L. Havel, J. Krejci, J. Corral Jaime, H. Chang, W.J. Geese, P. Bhagavatheeswaran, A.C. Chen, M.A. Socinski, I. CheckMate, First-Line Nivolumab in Stage IV or Recurrent Non-Small-Cell Lung Cancer, N Engl J Med 376(25) (2017) 2415-2426.

[24] T. Powles, D.F. McDermott, B. Rini, R.J. Motzer, M.B. Atkins, L. Fong, R.W. Joseph, S.K. Pal, A. Ravaud, S. Bracarda, C.S. Rodriguez, M. Maio, M. Gore, V. Grünwald, M. Staehler, J. Qiu, A. Thobhani, M. Huseni, C. Schiff, B. Escudier, LBA39IMmotion150: Novel radiological endpoints and updated data from a randomized phase II trial investigating atezolizumab (atezo) with or without bevacizumab (bev) vs sunitinib (sun) in untreated metastatic renal cell carcinoma (mRCC), Annals of Oncology 28(suppl_5) (2017) mdx440.033-mdx440.033.

[25] C. Hsu, S.H. Lee, S. Ejadi, C. Even, R.B. Cohen, C. Le Tourneau, J.M. Mehnert, A. Algazi, E.M.J. van Brummelen, S. Saraf, P. Thanigaimani, J.D. Cheng, A.R. Hansen, Safety and Antitumor Activity of Pembrolizumab in Patients With Programmed Death-Ligand 1-Positive Nasopharyngeal Carcinoma: Results of the KEYNOTE-028 Study, J Clin Oncol 35(36) (2017) 4050-4056.

[26] D.F. McDermott, M.B. Atkins, R.J. Motzer, B.I. Rini, B.J. Escudier, L. Fong, R.W. Joseph, S.K. Pal, M. Sznol, J.D. Hainsworth, W.M. Stadler, T.E. Hutson, A. Ravaud, S. Bracarda, C. Suarez, T.K. Choueiri, Y. Choi, M.A. Huseni, G.D. Fine, T. Powles, A phase II study of atezolizumab (atezo) with or without bevacizumab (bev) versus sunitinib (sun) in untreated metastatic renal cell carcinoma (mRCC) patients (pts), Journal of Clinical Oncology 35(6_suppl) (2017) 431-431.

[27] R.J. Motzer, B. Escudier, D.F. McDermott, S. George, H.J. Hammers, S. Srinivas, S.S. Tykodi, J.A. Sosman, G. Procopio, E.R. Plimack, D. Castellano, T.K. Choueiri, H. Gurney, F. Donskov, P. Bono, J. Wagstaff, T.C. Gauler, T. Ueda, Y. Tomita, F.A. Schutz, C. Kollmannsberger, J. Larkin, A. Ravaud, J.S. Simon, L.A. Xu, I.M. Waxman, P. Sharma, I. CheckMate, Nivolumab versus Everolimus in Advanced Renal-Cell Carcinoma, N Engl J Med 373(19) (2015) 1803-13.

[28] P. Sharma, M.K. Callahan, P. Bono, J. Kim, P. Spiliopoulou, E. Calvo, R.N. Pillai, P.A. Ott, F. de Braud, M. Morse, D.T. Le, D. Jaeger, E. Chan, C. Harbison, C.S. Lin, M. Tschaika, A. Azrilevich, J.E. Rosenberg, Nivolumab monotherapy in recurrent metastatic urothelial carcinoma (CheckMate 032): a multicentre, open-label, two-stage, multi-arm, phase 1/2 trial, Lancet Oncol 17(11) (2016) 1590-1598.

[29] A.V. Balar, D.E. Castellano, P.H. O'Donnell, P. Grivas, J. Vuky, T. Powles, E.R. Plimack, N.M. Hahn, R.D. Wit, L. Pang, M. Savage, R.F. Perini, S.M. Keefe, D.F. Bajorin, J. Bellmunt, Pembrolizumab as first-line therapy in cisplatin-ineligible advanced urothelial cancer: Results from the total KEYNOTE-052 study population, Journal of Clinical Oncology 35(6_suppl) (2017) 284-284.

[30] K. Hasegawa, K. Tamura, N. Katsumata, K. Matsumoto, S. Takahashi, H. Mukai, H. Nomura, H. Minami, Efficacy and safety of nivolumab (Nivo) in patients (pts) with advanced or recurrent uterine cervical or corpus cancers, Journal of Clinical Oncology 36(15_suppl) (2018) 5594-5594.

[31] A. Ribas, I. Puzanov, R. Dummer, D. Schadendorf, O. Hamid, C. Robert, F.S. Hodi, J. Schachter, A.C. Pavlick, K.D. Lewis, L.D. Cranmer, C.U. Blank, S.J. O'Day, P.A. Ascierto, A.K. Salama, K.A. Margolin, C. Loquai, T.K. Eigentler, T.C. Gangadhar, M.S. Carlino, S.S. Agarwala, S.J. Moschos, J.A. Sosman, S.M. Goldinger, R. Shapira-Frommer, R. Gonzalez, J.M. Kirkwood, J.D. Wolchok, A. Eggermont, X.N. Li, W. Zhou, A.M. Zernhelt, J. Lis, S. Ebbinghaus, S.P. Kang, A. Daud, Pembrolizumab versus investigator-choice chemotherapy for ipilimumab-refractory melanoma (KEYNOTE-002): a randomised, controlled, phase 2 trial, Lancet Oncol 16(8) (2015) 908-18.

[32] S. Adams, P. Schmid, H.S. Rugo, E.P. Winer, D. Loirat, A. Awada, D.W. Cescon, H. Iwata, M. Campone, R. Nanda, R. Hui, G. Curigliano, D. Toppmeyer, J. O'Shaughnessy, S. Loi, S. Paluch-Shimon, D. Card, J. Zhao, V. Karantza, J. Cortes, Phase 2 study of pembrolizumab (pembro) monotherapy for previously treated metastatic triple-negative breast cancer (mTNBC): KEYNOTE-086 cohort A, Journal of Clinical Oncology 35(15_suppl) (2017) 1008-1008.

[33] M. Garassino, N. Rizvi, B. Besse, P. Jänne, D. Christoph, S. Peters, C.K. Toh, T. Kurata, E. Carcereny Costa, M. Koczywas, E. Felip, J. Chaft, J. Qiu, M. Kowanetz, S. Coleman, S. Mocci, A. Sandler, S. Gettinger, M. Johnson, OA03.02 Atezolizumab as 1L Therapy for Advanced NSCLC in PD-L1&#x2013;Selected Patients: Updated ORR, PFS and OS Data&#xa0;from the BIRCH Study, Journal of Thoracic Oncology 12(1) (2017) S251-S252.

[34] S. Gettinger, N.A. Rizvi, L.Q. Chow, H. Borghaei, J. Brahmer, N. Ready, D.E. Gerber, F.A. Shepherd, S. Antonia, J.W. Goldman, R.A. Juergens, S.A. Laurie, F.E. Nathan, Y. Shen, C.T. Harbison, M.D. Hellmann, Nivolumab Monotherapy for First-Line Treatment of Advanced Non-Small-Cell Lung Cancer, J Clin Oncol 34(25) (2016) 2980-7.

[35] E.B. Garon, L. Gandhi, N. Rizvi, R. Hui, A.S. Balmanoukian, A. Patnaik, J.P. Eder, G.R. Blumenshein, C. Aggarwal, J.C. Soria, M.A. Ahn, M.A. Gubens, S.S. Ramalingam, E. Johnson, H. Arkenau, G.M. Lubiniecki, J. Zhang, R.Z. Rutledge, K. Emancipator, N. Leighl, LBA43ANTITUMOR ACTIVITY OF PEMBROLIZUMAB (PEMBRO; MK-3475) AND CORRELATION WITH PROGRAMMED DEATH LIGAND 1 (PD-L1) EXPRESSION IN A POOLED ANALYSIS OF PATIENTS (PTS) WITH ADVANCED NON–SMALL CELL LUNG CARCINOMA (NSCLC), Annals of Oncology 25(suppl_4) (2014) mdu438.51-mdu438.51.

[36] K. Muro, H.C. Chung, V. Shankaran, R. Geva, D. Catenacci, S. Gupta, J.P. Eder, T. Golan, D.T. Le, B. Burtness, A.J. McRee, C.C. Lin, K. Pathiraja, J. Lunceford, K. Emancipator, J. Juco, M. Koshiji, Y.J. Bang, Pembrolizumab for patients with PD-L1-positive advanced gastric cancer (KEYNOTE-012): a multicentre, open-label, phase 1b trial, Lancet Oncol 17(6) (2016) 717-726.

[37] S. Peters, S. Gettinger, M.L. Johnson, P.A. Janne, M.C. Garassino, D. Christoph, C.K. Toh, N.A. Rizvi, J.E. Chaft, E. Carcereny Costa, J.D. Patel, L.Q.M. Chow, M. Koczywas, C. Ho, M. Fruh, M. van den Heuvel, J. Rothenstein, M. Reck, L. Paz-Ares, F.A. Shepherd, T. Kurata, Z. Li, J. Qiu, M. Kowanetz, S. Mocci, G. Shankar, A. Sandler, E. Felip, Phase II Trial of Atezolizumab As First-Line or Subsequent Therapy for Patients With Programmed Death-Ligand 1-Selected Advanced Non-Small-Cell Lung Cancer (BIRCH), J Clin Oncol 35(24) (2017) 2781-2789.

[38] J. Bellmunt, R. de Wit, D.J. Vaughn, Y. Fradet, J.L. Lee, L. Fong, N.J. Vogelzang, M.A. Climent, D.P. Petrylak, T.K. Choueiri, A. Necchi, W. Gerritsen, H. Gurney, D.I. Quinn, S. Culine, C.N. Sternberg, Y. Mai, C.H. Poehlein, R.F. Perini, D.F. Bajorin, K.-. Investigators, Pembrolizumab as Second-Line Therapy for Advanced Urothelial Carcinoma, N Engl J Med 376(11) (2017) 1015-1026.

[39] B.B.Y. Ma, W.T. Lim, B.C. Goh, E.P. Hui, K.W. Lo, A. Pettinger, N.R. Foster, J.W. Riess, M. Agulnik, A.Y.C. Chang, A. Chopra, J.A. Kish, C.H. Chung, D.R. Adkins, K.J. Cullen, B.J. Gitlitz, D.W. Lim, K.F. To, K.C.A. Chan, Y.M.D. Lo, A.D. King, C. Erlichman, J. Yin, B.A. Costello, A.T.C. Chan, Antitumor Activity of Nivolumab in Recurrent and Metastatic Nasopharyngeal Carcinoma: An International, Multicenter Study of the Mayo Clinic Phase 2 Consortium (NCI-9742), J Clin Oncol 36(14) (2018) 1412-1418.

[40] A.B. El-Khoueiry, B. Sangro, T. Yau, T.S. Crocenzi, M. Kudo, C. Hsu, T.Y. Kim, S.P. Choo, J. Trojan, T.H.R. Welling, T. Meyer, Y.K. Kang, W. Yeo, A. Chopra, J. Anderson, C. Dela Cruz, L. Lang, J. Neely, H. Tang, H.B. Dastani, I. Melero, Nivolumab in patients with advanced hepatocellular carcinoma (CheckMate 040): an open-label, non-comparative, phase 1/2 dose escalation and expansion trial, Lancet 389(10088) (2017) 2492-2502.

[41] E.W. Alley, J. Lopez, A. Santoro, A. Morosky, S. Saraf, B. Piperdi, E. van Brummelen, Clinical safety and activity of pembrolizumab in patients with malignant pleural mesothelioma (KEYNOTE-028): preliminary results from a non-randomised, open-label, phase 1b trial, Lancet Oncol 18(5) (2017) 623-630.

[42] H. Borghaei, L. Paz-Ares, L. Horn, D.R. Spigel, M. Steins, N.E. Ready, L.Q. Chow, E.E. Vokes, E. Felip, E. Holgado, F. Barlesi, M. Kohlhaufl, O. Arrieta, M.A. Burgio, J. Fayette, H. Lena, E. Poddubskaya, D.E. Gerber, S.N. Gettinger, C.M. Rudin, N. Rizvi, L. Crino, G.R. Blumenschein, Jr., S.J. Antonia, C. Dorange, C.T. Harbison, F. Graf Finckenstein, J.R. Brahmer, Nivolumab versus Docetaxel in Advanced Nonsquamous Non-Small-Cell Lung Cancer, N Engl J Med 373(17) (2015) 1627-39.

[43] C.F. Verschraegen, F. Chen, D.R. Spigel, N. Iannotti, E.F. McClay, C.H. Redfern, J. Bennouna, M.H. Taylor, H. Kaufman, K. Kelly, M. Bajars, A.v. Heydebreck, J.-M. Cuillerot, G.H.M. Jerusalem, Avelumab (MSB0010718C; anti-PD-L1) as a first-line treatment for patients with advanced NSCLC from the JAVELIN Solid Tumor phase 1b trial: Safety, clinical activity, and PD-L1 expression, Journal of Clinical Oncology 34(15_suppl) (2016) 9036-9036.

[44] H.C. Chung, J.A. Lopez-Martin, S.C.-H. Kao, W.H. Miller, W. Ros, B. Gao, A. Marabelle, M. Gottfried, A. Zer, J.-P. Delord, N. Penel, S.I. Jalal, L. Xu, S. Zeigenfuss, S.K. Pruitt, S.A. Piha-Paul, Phase 2 study of pembrolizumab in advanced small-cell lung cancer (SCLC): KEYNOTE-158, Journal of Clinical Oncology 36(15_suppl) (2018) 8506-8506.

[45] R. Nanda, L.Q. Chow, E.C. Dees, R. Berger, S. Gupta, R. Geva, L. Pusztai, K. Pathiraja, G. Aktan, J.D. Cheng, V. Karantza, L. Buisseret, Pembrolizumab in Patients With Advanced Triple-Negative Breast Cancer: Phase Ib KEYNOTE-012 Study, J Clin Oncol 34(21) (2016) 2460-7.

[46] G. Zalcman, J. Mazieres, L. Greillier, S. Lantuejoul, P. Dô, O. Bylicki, I. Monnet, R. Corre, C. Audigier-Valette, M. Locatelli-Sanchez, O. Molinier, L. Thiberville, T. Urban, D. Planchard, C. Ligeza-Poisson, E. Amour, F. Morin, D. Moro-Sibilot, A. Scherpereel, LBA58_PRSecond or 3rd line nivolumab (Nivo) versus nivo plus ipilimumab (Ipi) in malignant pleural mesothelioma (MPM) patients: Updated results of the IFCT-1501 MAPS2 randomized phase 2 trial, Annals of Oncology 28(suppl_5) (2017) mdx440.074-mdx440.074.

[47] T.Y. Seiwert, B. Burtness, R. Mehra, J. Weiss, R. Berger, J.P. Eder, K. Heath, T. McClanahan, J. Lunceford, C. Gause, J.D. Cheng, L.Q. Chow, Safety and clinical activity of pembrolizumab for treatment of recurrent or metastatic squamous cell carcinoma of the head and neck (KEYNOTE-012): an open-label, multicentre, phase 1b trial, Lancet Oncol 17(7) (2016) 956-65.

[48] A.B. Apolo, J.R. Infante, A. Balmanoukian, M.R. Patel, D. Wang, K. Kelly, A.E. Mega, C.D. Britten, A. Ravaud, A.C. Mita, H. Safran, T.E. Stinchcombe, M. Srdanov, A.B. Gelb, M. Schlichting, K. Chin, J.L. Gulley, Avelumab, an Anti–Programmed Death-Ligand 1 Antibody, In Patients With Refractory Metastatic Urothelial Carcinoma: Results From a Multicenter, Phase Ib Study, Journal of Clinical Oncology 35(19) (2017) 2117-2124.

[49] Y.J. Bang, T. Doi, F.D. Braud, S. Piha-Paul, A. Hollebecque, A.R.A. Razak, C.C. Lin, P.A. Ott, A.R. He, S.S. Yuan, M. Koshiji, B. Lam, R. Aggarwal, 525 Safety and efficacy of pembrolizumab (MK-3475) in patients (pts) with advanced biliary tract cancer: Interim results of KEYNOTE-028, European Journal of Cancer 51 (2015) S112.

[50] J.-S. Frenel, C.L. Tourneau, B.H. O'Neil, P.A. Ott, S.A. Piha-Paul, C.A. Gomez-Roca, E.V. Brummelen, H.S. Rugo, S. Thomas, S. Saraf, M. Chen, A. Varga, Pembrolizumab in patients with advanced cervical squamous cell cancer: Preliminary results from the phase Ib KEYNOTE-028 study, Journal of Clinical Oncology 34(15_suppl) (2016) 5515-5515.

[51] P.A. Ott, S.A. Piha-Paul, P. Munster, M.J. Pishvaian, E.M.J. van Brummelen, R.B. Cohen, C. Gomez-Roca, S. Ejadi, M. Stein, E. Chan, M. Simonelli, A. Morosky, S. Saraf, K. Emancipator, M. Koshiji, J. Bennouna, Safety and antitumor activity of the anti-PD-1 antibody pembrolizumab in patients with recurrent carcinoma of the anal canal, Ann Oncol 28(5) (2017) 1036-1041.

[52] A.X. Zhu, R.S. Finn, S. Cattan, J. Edeline, S. Ogasawara, D.H. Palmer, C. Verslype, V. Zagonel, O. Rosmorduc, A. Vogel, D. Sarker, G. Verset, S.L. Chan, J.J. Knox, B. Daniele, S. Ebbinghaus, J. Ma, A.B. Siegel, A.-L. Cheng, M. Kudo, KEYNOTE-224: Pembrolizumab in patients with advanced hepatocellular carcinoma previously treated with sorafenib, Journal of Clinical Oncology 36(4_suppl) (2018) 209-209.

[53] J. Bauml, T.Y. Seiwert, D.G. Pfister, F. Worden, S.V. Liu, J. Gilbert, N.F. Saba, J. Weiss, L. Wirth, A. Sukari, H. Kang, M.K. Gibson, E. Massarelli, S. Powell, A. Meister, X. Shu, J.D. Cheng, R. Haddad, Pembrolizumab for Platinum- and Cetuximab-Refractory Head and Neck Cancer: Results From a Single-Arm, Phase II Study, J Clin Oncol 35(14) (2017) 1542-1549.

[54] K. Shitara, M. Özgüroğlu, Y.J. Bang, M. Di Bartolomeo, M. Mandalà, M.H. Ryu, L. Fornaro, T. Olesiński, C. Caglevic, H. Chung, K. Muro, E. Gökkurt, W. Mansoor, R. McDermott, E. Schacham-Shmueli, X. Chen, S.P. Kang, C. Mayo, A. Ohtsu, C. Fuchs, LBA-005KEYNOTE-061: Phase 3 study of pembrolizumab vs paclitaxel for previously treated advanced gastric or gastroesophageal junction (G/GEJ) cancer, Annals of Oncology 29(suppl_5) (2018) mdy208.004-mdy208.004.

[55] J. Hamanishi, M. Mandai, T. Ikeda, M. Minami, A. Kawaguchi, T. Murayama, M. Kanai, Y. Mori, S. Matsumoto, S. Chikuma, N. Matsumura, K. Abiko, T. Baba, K. Yamaguchi, A. Ueda, Y. Hosoe, S. Morita, M. Yokode, A. Shimizu, T. Honjo, I. Konishi, Safety and Antitumor Activity of Anti-PD-1 Antibody, Nivolumab, in Patients With Platinum-Resistant Ovarian Cancer, J Clin Oncol 33(34) (2015) 4015-22.

[56] C.S. Fuchs, T. Doi, R.W.-J. Jang, K. Muro, T. Satoh, M. Machado, W. Sun, S.I. Jalal, M.A. Shah, J.-P. Metges, M. Garrido, T. Golan, M. Mandala, Z.A. Wainberg, D.V.T. Catenacci, Y.-J. Bang, J. Wang, M. Koshiji, R.P. Dalal, H.H. Yoon, KEYNOTE-059 cohort 1: Efficacy and safety of pembrolizumab (pembro) monotherapy in patients with previously treated advanced gastric cancer, Journal of Clinical Oncology 35(15_suppl) (2017) 4003-4003.

[57] E.E. Cohen, K. Harrington, C. Le Tourneau, J. Dinis, L. Licitra, M.J. Ahn, A. Soria, J.P. Machiels, N. Mach, R. Mehra, B. Burtness, Y. Wang, A.J. Tuozzo, R. Swaby, D. Soulieres, LBA45_PRPembrolizumab (pembro) vs standard of care (SOC) for recurrent or metastatic head and neck squamous cell carcinoma (R/M HNSCC): Phase 3 KEYNOTE-040 trial, 2017.

[58] D. Soulieres, E. Cohen, C.L. Tourneau, J. Dinis, L. Licitra, M.-J. Ahn, A. Soria, J.-P. Machiels, N. Mach, R. Mehra, B. Burtness, P. Zhang, J. Cheng, R. Swaby, K.J. Harrington, Abstract CT115: Updated survival results of the KEYNOTE-040 study of pembrolizumab vs standard-of-care chemotherapy for recurrent or metastatic head and neck squamous cell carcinoma, Cancer Research 78(13 Supplement) (2018) CT115-CT115.

[59] A. Rittmeyer, F. Barlesi, D. Waterkamp, K. Park, F. Ciardiello, J. von Pawel, S.M. Gadgeel, T. Hida, D.M. Kowalski, M.C. Dols, D.L. Cortinovis, J. Leach, J. Polikoff, C. Barrios, F. Kabbinavar, O.A. Frontera, F. De Marinis, H. Turna, J.S. Lee, M. Ballinger, M. Kowanetz, P. He, D.S. Chen, A. Sandler, D.R. Gandara, O.A.K.S. Group, Atezolizumab versus docetaxel in patients with previously treated non-small-cell lung cancer (OAK): a phase 3, open-label, multicentre randomised controlled trial, Lancet 389(10066) (2017) 255-265.

[60] R.L. Ferris, G. Blumenschein, Jr., J. Fayette, J. Guigay, A.D. Colevas, L. Licitra, K. Harrington, S. Kasper, E.E. Vokes, C. Even, F. Worden, N.F. Saba, L.C. Iglesias Docampo, R. Haddad, T. Rordorf, N. Kiyota, M. Tahara, M. Monga, M. Lynch, W.J. Geese, J. Kopit, J.W. Shaw, M.L. Gillison, Nivolumab for Recurrent Squamous-Cell Carcinoma of the Head and Neck, N Engl J Med 375(19) (2016) 1856-1867.

[61] P.A. Ott, Y.J. Bang, D. Berton-Rigaud, E. Elez, M.J. Pishvaian, H.S. Rugo, I. Puzanov, J.M. Mehnert, K.L. Aung, J. Lopez, M. Carrigan, S. Saraf, M. Chen, J.C. Soria, Safety and Antitumor Activity of Pembrolizumab in Advanced Programmed Death Ligand 1-Positive Endometrial Cancer: Results From the KEYNOTE-028 Study, J Clin Oncol 35(22) (2017) 2535-2541.

[62] A. Hansen, C. Massard, P.A. Ott, N. Haas, J. Lopez, S. Ejadi, J. Wallmark, B. Keam, J.P. Delord, R. Aggarwal, M. Gould, P. Qiu, S. Saraf, S. Keefe, S.A. Piha-Paul, Pembrolizumab for patients with advanced prostate adenocarcinoma: Preliminary results from the KEYNOTE-028 study, Annals of Oncology 27(suppl_6) (2016) 725PD-725PD.

[63] H.C. Chung, J.H.M. Schellens, J.-P. Delord, R. Perets, A. Italiano, R. Shapira-Frommer, L. Manzuk, S.A. Piha-Paul, J. Wang, S. Zeigenfuss, S.K. Pruitt, A. Marabelle, Pembrolizumab treatment of advanced cervical cancer: Updated results from the phase 2 KEYNOTE-158 study, Journal of Clinical Oncology 36(15_suppl) (2018) 5522-5522.

[64] H.S. Rugo, J.P. Delord, S.A. Im, P.A. Ott, S.A. Piha-Paul, P.L. Bedard, J. Sachdev, C.L. Tourneau, E.M.J. van Brummelen, A. Varga, R. Salgado, S. Loi, S. Saraf, D. Pietrangelo, V. Karantza, A.R. Tan, Safety and Antitumor Activity of Pembrolizumab in Patients with Estrogen Receptor-Positive/Human Epidermal Growth Factor Receptor 2-Negative Advanced Breast Cancer, Clin Cancer Res 24(12) (2018) 2804-2811.

[65] J.M. Mehnert, H.S. Rugo, B.H. O'Neil, A. Santoro, J.H.M. Schellens, R.B. Cohen, T. Doi, P.A. Ott, M.J. Pishvaian, I. Puzanov, K.L. Aung, C. Hsu, C. Le Tourneau, J.C. Soria, E. Elez, K. Tamura, M. Gould, G. Zhao, K. Stein, S.A. Piha-Paul, 427OPembrolizumab for patients with PD-L1–positive advanced carcinoid or pancreatic neuroendocrine tumors: Results from the KEYNOTE-028 study, Annals of Oncology 28(suppl_5) (2017) mdx368-mdx368.

[66] M.A. Shah, T. Kojima, P.C. Enzinger, D. Hochhauser, J. Raimbourg, A. Hollebecque, F. Lordick, S.-B. Kim, M. Tajika, H.T. Kim, A.C. Lockhart, H.-T. Arkenau, F.E. Hajbi, M. Gupta, P. Pfeiffer, Q. Liu, J. Lunceford, S.P. Kang, P. Bhagia, K. Kato, Pembrolizumab for patients with previously treated metastatic adenocarcinoma or squamous cell carcinoma of the esophagus: Phase 2 KEYNOTE-180 study, Journal of Clinical Oncology 36(15_suppl) (2018) 4049-4049.

[67] J.L. Gulley, A. Rajan, D.R. Spigel, N. Iannotti, J. Chandler, D.J.L. Wong, J. Leach, W.J. Edenfield, D. Wang, H.J. Grote, A.V. Heydebreck, K. Chin, J.M. Cuillerot, K. Kelly, Avelumab for patients with previously treated metastatic or recurrent non-small-cell lung cancer (JAVELIN Solid Tumor): dose-expansion cohort of a multicentre, open-label, phase 1b trial, Lancet Oncol 18(5) (2017) 599-610.

[68] C.S. Fuchs, T. Doi, R.W. Jang, K. Muro, T. Satoh, M. Machado, W. Sun, S.I. Jalal, M.A. Shah, J.P. Metges, M. Garrido, T. Golan, M. Mandala, Z.A. Wainberg, D.V. Catenacci, A. Ohtsu, K. Shitara, R. Geva, J. Bleeker, A.H. Ko, G. Ku, P. Philip, P.C. Enzinger, Y.J. Bang, D. Levitan, J. Wang, M. Rosales, R.P. Dalal, H.H. Yoon, Safety and Efficacy of Pembrolizumab Monotherapy in Patients With Previously Treated Advanced Gastric and Gastroesophageal Junction Cancer: Phase 2 Clinical KEYNOTE-059 Trial, JAMA oncology 4(5) (2018) e180013.

[69] A. Varga, S.A. Piha-Paul, P.A. Ott, J.M. Mehnert, D. Berton-Rigaud, A. Morosky, G.Q. Zhao, R.A. Rangwala, D. Matei, Pembrolizumab in patients (pts) with PD-L1–positive (PD-L1+) advanced ovarian cancer: Updated analysis of KEYNOTE-028, Journal of Clinical Oncology 35(15_suppl) (2017) 5513-5513.

[70] R.B. Cohen, J.-P. Delord, T. Doi, S.A. Piha-Paul, S.V. Liu, J. Gilbert, A.P. Algazi, S. Cresta, R.-L. Hong, C.L. Tourneau, D. Day, A. Varga, E. Elez, J.M. Wallmark, S. Saraf, A. Morosky, J.D. Cheng, B. Keam, Preliminary results for the advanced salivary gland carcinoma cohort of the phase 1b KEYNOTE-028 study of pembrolizumab, Journal of Clinical Oncology 34(15_suppl) (2016) 6017-6017.

[71] Y.-K. Kang, T. Satoh, M.-H. Ryu, Y. Chao, K. Kato, H.C. Chung, J.-S. Chen, K. Muro, W.K. Kang, T. Yoshikawa, S.C. Oh, T. Tamura, K.-W. Lee, N. Boku, L.-T. Chen, Nivolumab (ONO-4538/BMS-936558) as salvage treatment after second or later-line chemotherapy for advanced gastric or gastro-esophageal junction cancer (AGC): A double-blinded, randomized, phase III trial, Journal of Clinical Oncology 35(4_suppl) (2017) 2-2.

[72] C. Le Tourneau, C. Zarwan, C. Hoimes, D.J. Wong, S. Bauer, M. Wermke, H.J. Grote, A. von Heydebreck, K. Chin, J. Gulley, 913PAvelumab in patients with metastatic adrenocortical carcinoma (mACC): Results from the JAVELIN solid tumor trial, Annals of Oncology 28(suppl_5) (2017) mdx371.067-mdx371.067.

[73] M.L. Disis, M.R. Patel, S. Pant, E.P. Hamilton, A.C. Lockhart, K. Kelly, J.T. Beck, M.S. Gordon, G.J. Weiss, M.H. Taylor, J. Chaves, A.C. Mita, K.M. Chin, A.v. Heydebreck, J.-M. Cuillerot, J.L. Gulley, Avelumab (MSB0010718C; anti-PD-L1) in patients with recurrent/refractory ovarian cancer from the JAVELIN Solid Tumor phase Ib trial: Safety and clinical activity, Journal of Clinical Oncology 34(15_suppl) (2016) 5533-5533.

[74] R. Hassan, A. Thomas, J.J. Nemunaitis, M.R. Patel, J. Bennouna, F. Chen, J.-P. Delord, A. Dowlati, M.H. Taylor, J.D. Powderly, U.N. Vaishampayan, C.F. Verschraegen, H.J. Grote, A.v. Heydebreck, K.M. Chin, J.L. Gulley, Avelumab in patients with previously treated mesothelioma: Updated phase 1b results from the JAVELIN Solid Tumor trial, Journal of Clinical Oncology 36(5_suppl) (2018) 166-166.

[75] J.M. Mehnert, A. Varga, M. Brose, R.R. Aggarwal, C.-C. Lin, A. Prawira, F.d. Braud, K. Tamura, T. Doi, S.A. Piha-Paul, J. Gilbert, S. Saraf, P. Thanigaimani, J.D. Cheng, B. Keam, Pembrolizumab for advanced papillary or follicular thyroid cancer: preliminary results from the phase 1b KEYNOTE-028 study, Journal of Clinical Oncology 34(15_suppl) (2016) 6091-6091.

[76] H.C. Chung, H.-T. Arkenau, J. Lee, S.Y. Rha, D.-Y. Oh, L. Wyrwicz, Y.-K. Kang, K.-W. Lee, T.M. Bauer, S.S. Lee, M. Kemeny, U. Keilholz, B. Melichar, A. Mita, R. Plummer, D. Smith, A.B. Gelb, H. Xiong, J. Hong, V. Chand, H. Safran, Abstract CT111: Avelumab (anti-PD-L1) as first-line maintenance (1L mn) or second-line (2L) therapy in patients with advanced gastric or gastroesophageal junction cancer (GC/GEJC): updated phase Ib results from the JAVELIN Solid Tumor trial, Cancer Research 78(13 Supplement) (2018) CT111-CT111.

[77] L.Y. Dirix, I. Takacs, G. Jerusalem, P. Nikolinakos, H.T. Arkenau, A. Forero-Torres, R. Boccia, M.E. Lippman, R. Somer, M. Smakal, L.A. Emens, B. Hrinczenko, W. Edenfield, J. Gurtler, A. von Heydebreck, H.J. Grote, K. Chin, E.P. Hamilton, Avelumab, an anti-PD-L1 antibody, in patients with locally advanced or metastatic breast cancer: a phase 1b JAVELIN Solid Tumor study, Breast Cancer Res Treat 167(3) (2018) 671-686.

[78] J.S.D. Bono, J.C. Goh, K. Ojamaa, J.M.P. Rodriguez, C.G. Drake, C.J. Hoimes, H. Wu, C.H. Poehlein, E.S. Antonarakis, KEYNOTE-199: Pembrolizumab (pembro) for docetaxel-refractory metastatic castration-resistant prostate cancer (mCRPC), Journal of Clinical Oncology 36(15_suppl) (2018) 5007-5007.

[79] B.H. O'Neil, J.M. Wallmark, D. Lorente, E. Elez, J. Raimbourg, C. Gomez-Roca, S. Ejadi, S.A. Piha-Paul, M.N. Stein, A.R. Abdul Razak, K. Dotti, A. Santoro, R.B. Cohen, M. Gould, S. Saraf, K. Stein, S.W. Han, Safety and antitumor activity of the anti-PD-1 antibody pembrolizumab in patients with advanced colorectal carcinoma, PLoS ONE 12(12) (2017) e0189848.
